# Supplementary material for: Structural basis of human Mediator recruitment by the phosphorylated transcription factor Elk-1
Source: Nat Commun. 2025 Apr 22;16:3772. doi: 10.1038/s41467-025-59014-8 (PMC12015215; doi:10.1038/s41467-025-59014-8)
Supplement: Supplementary file 1 — Supplementary Information [file 41467_2025_59014_MOESM1_ESM.pdf]

Supplementary Figures

Supplementary Fig. 1: Overlay of MED23 crystal structure and Mediator complex cryo-EM structures

Supplementary Fig. 2: MED23 cryo-EM data collection and processing

Supplementary Fig. 3: 3 Å resolution Cryo-EM map of MED23

Supplementary Fig. 4: MED23 HEAT-repeats

Supplementary Fig. 5: Phosphorylation of Elk-1

Supplementary Fig. 6: $MED23^{Elk-1}$ cryo-EM data collection and processing

Supplementary Fig. 7: 3 Å resolution Cryo-EM map of $MED23^{Elk-1}$

Supplementary Fig. 8: Contribution of Elk-1 phosphorylation to MED23 binding

Supplementary Fig. 9: Global movement of helix H19 in HR2 necessary for MED23 to accommodate F378-Elk-1

Supplementary Fig. 10: H613 and H614 double conformations

Supplementary Fig. 11: Structure of Mediator-bound preinitiation complex

Supplementary Table

Supplementary Table 1. Plasmids, antibodies and reagents

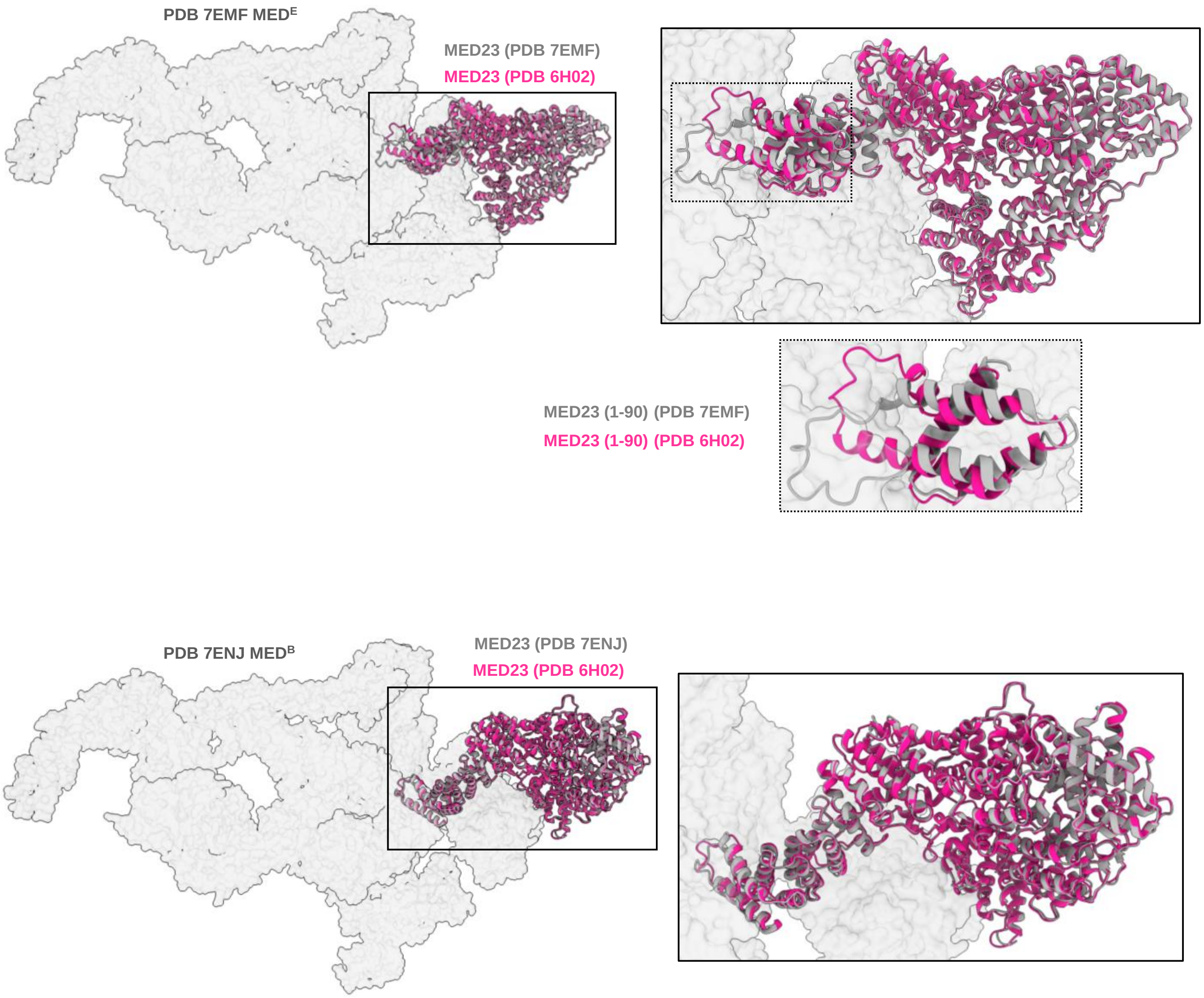

<u>Supplementary Fig. 1:</u> Overlay of MED23 crystal structure and Mediator complex cryo-EM structures. Superimposition of the Tail-extended (MED^E PDB 7EMF) or Tail-bent (MED^B PDB 7ENJ) Mediator complex cryo-EM structures in surface representation with MED23 crystal structure (PDB 6H02). Expanded view displays MED23 superimposed structures in ribbon with MED23 subunit from Mediator complex colored in light gray and MED23 crystal structure colored in deep pink. The structure of MED23 is conserved and superimposable, except for the N-terminal 1-50 region shown in close-up view (region 1-90) indicated by a black dashed box.

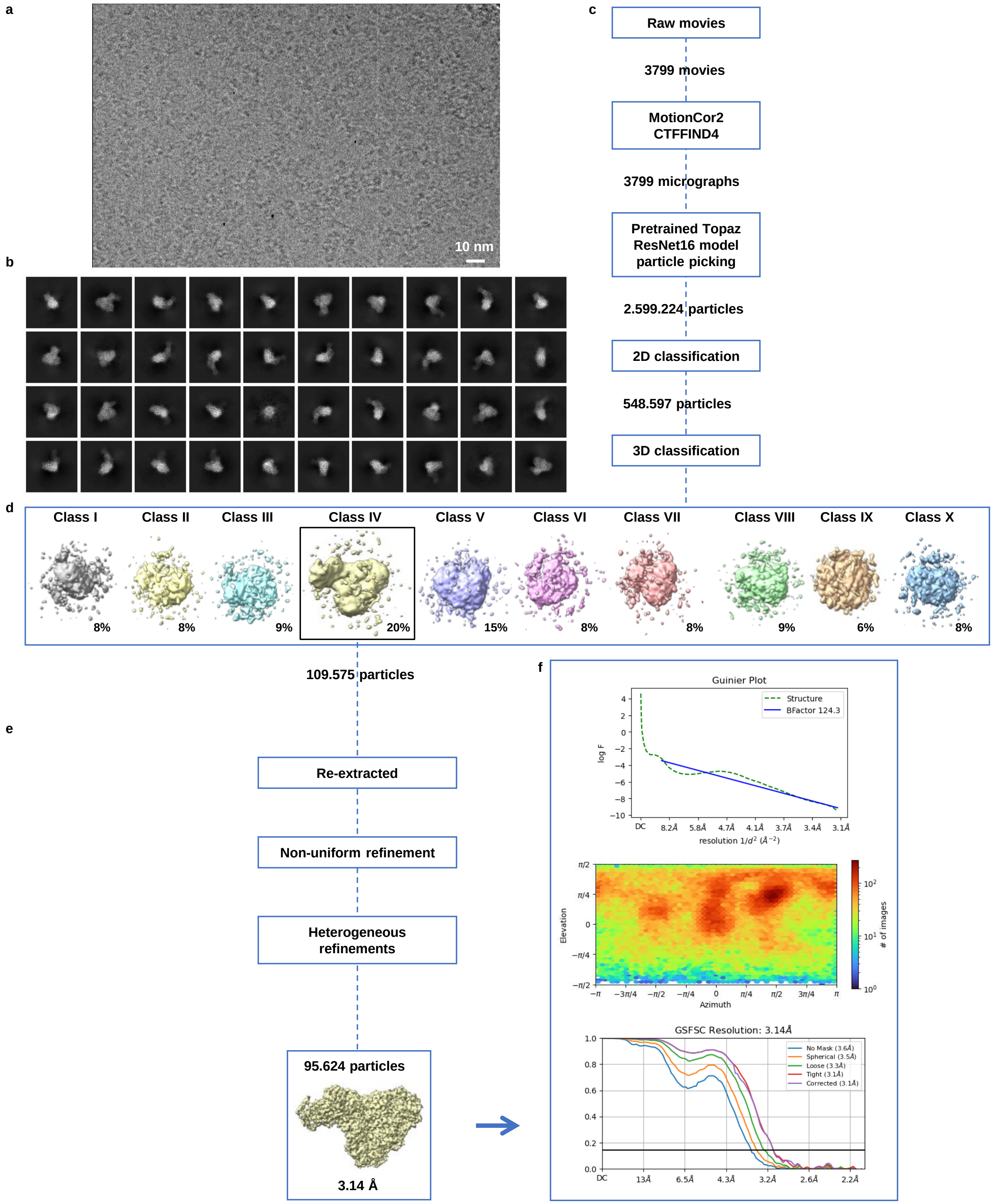

<u>Supplementary Fig. 2:</u> MED23 cryo-EM data collection and processing. a, Representative cryo-electron micrograph of MED23 sample. **b,** class averages after reference-free 2D classification generated with cryoSparc. Box size corresponds to 300 Å. **c,** Processing tree. The number of selected particles for each step is indicated. **d,** 109.575 particle images were assigned to a 3D-class (class IV) showing density for MED23 entity. **e,** A final reconstruction at 3.1 Å resolution was obtained by non-uniform refinement and rounds of heterogeneous refinements. **f**, Guinier plot used for local B-factor estimation, angular distribution plot after reconstruction with cryoSparc and Fourier shell correlation plot for the focused refined map.

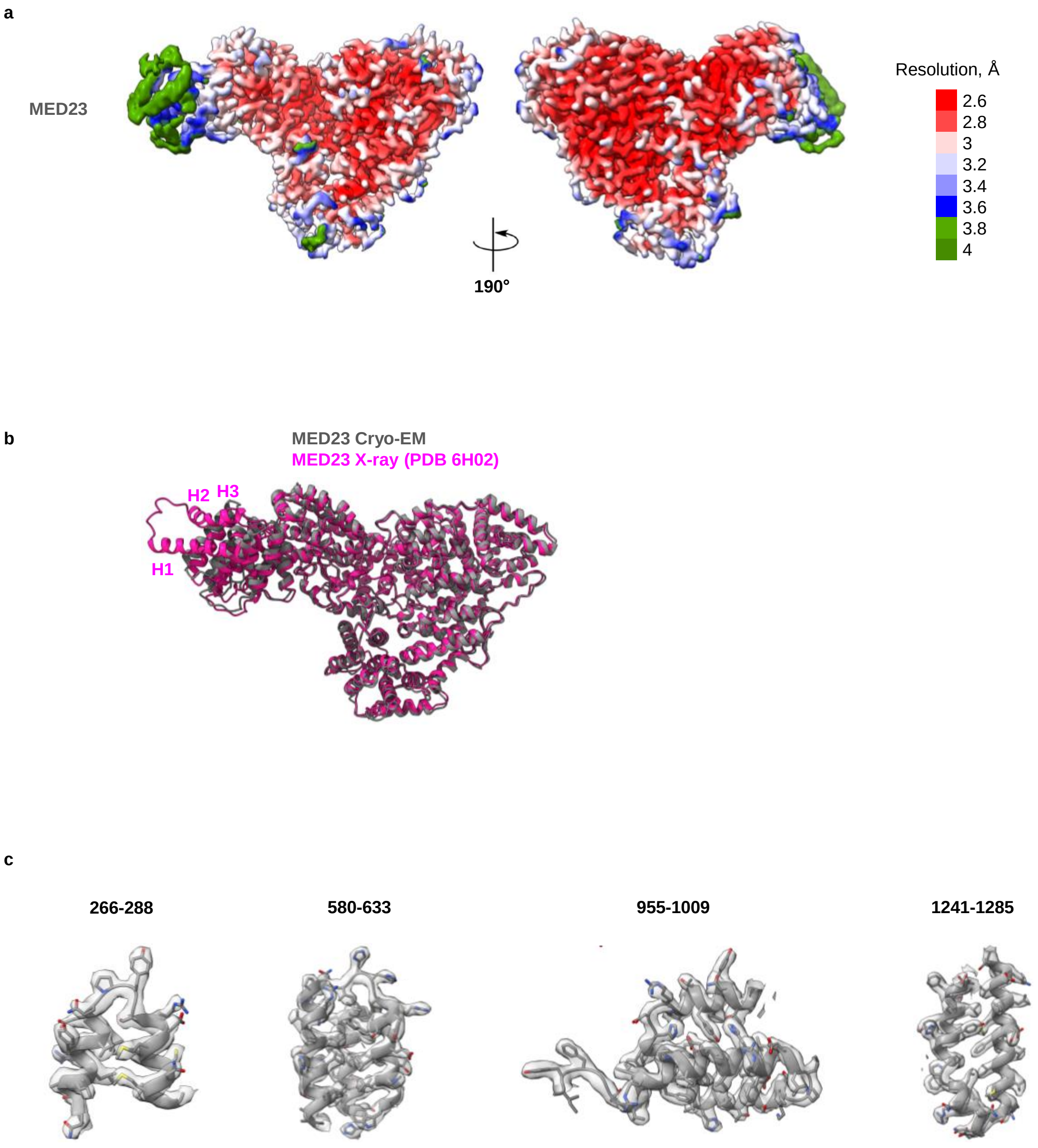

<u>Supplementary Fig. 3:</u> 3 Å resolution Cryo-EM map of MED23. a, Local resolution of MED23 with a 0.5 threshold for local FSC resolution estimation calculated in cryoSparc. The cryo-EM density surface is colored according to the resolution estimates, ranging from 2.6 Å (red) to 4 Å (green) depicted from the concave view and rotated by 190° (convex view). MED23 is well resolved below 3 Å, except for small region in loops and HR1 region. **b,** Superimposition of MED23 (in gray) with MED23 crystal structure (PDB 6H02 in deep pink). Residues 1-50 that contains the three N-terminal helices (H1 to H3) only showed blobs of density and thus have not been included in the model. **c,** Map-model fit of selected regions in surface representation for MED23. Side chains are represented in stick.

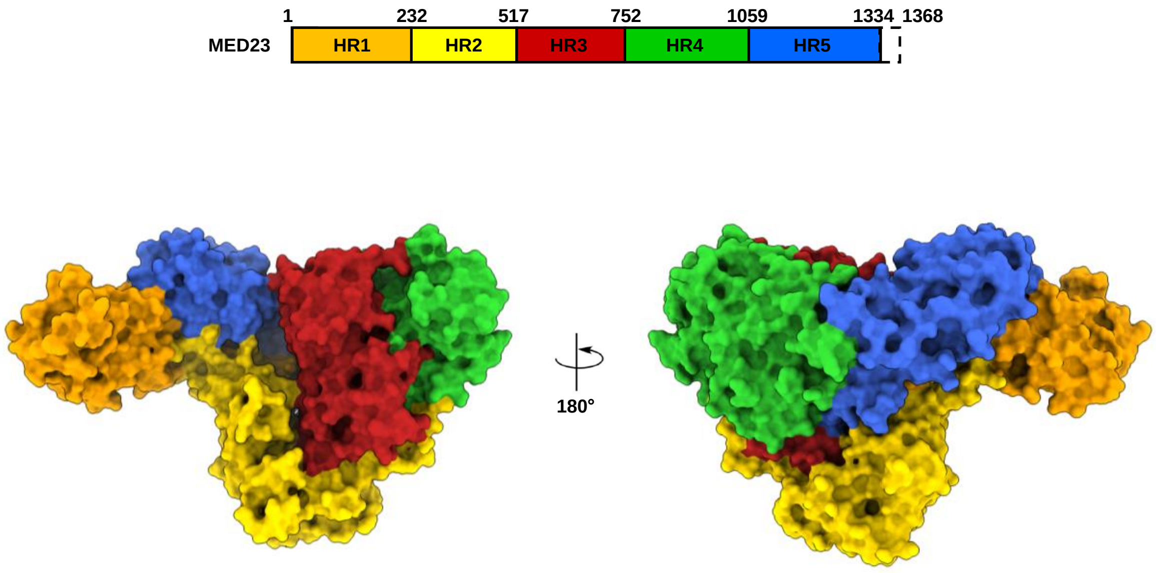

<u>Supplementary Fig. 4:</u> MED23 HEAT-repeats. Model of MED23 in concave and convex views related to Figs. 1b,c. Domains organization of MED23-HEAT-repeat boundaries (HR1 to HR5) are indicated. MED23 is fully folded with the exception of the last 30 amino acids indicated by a black dashed box. The individual HEAT-repeat HR1 to HR5 are represented in surface and color-coded.

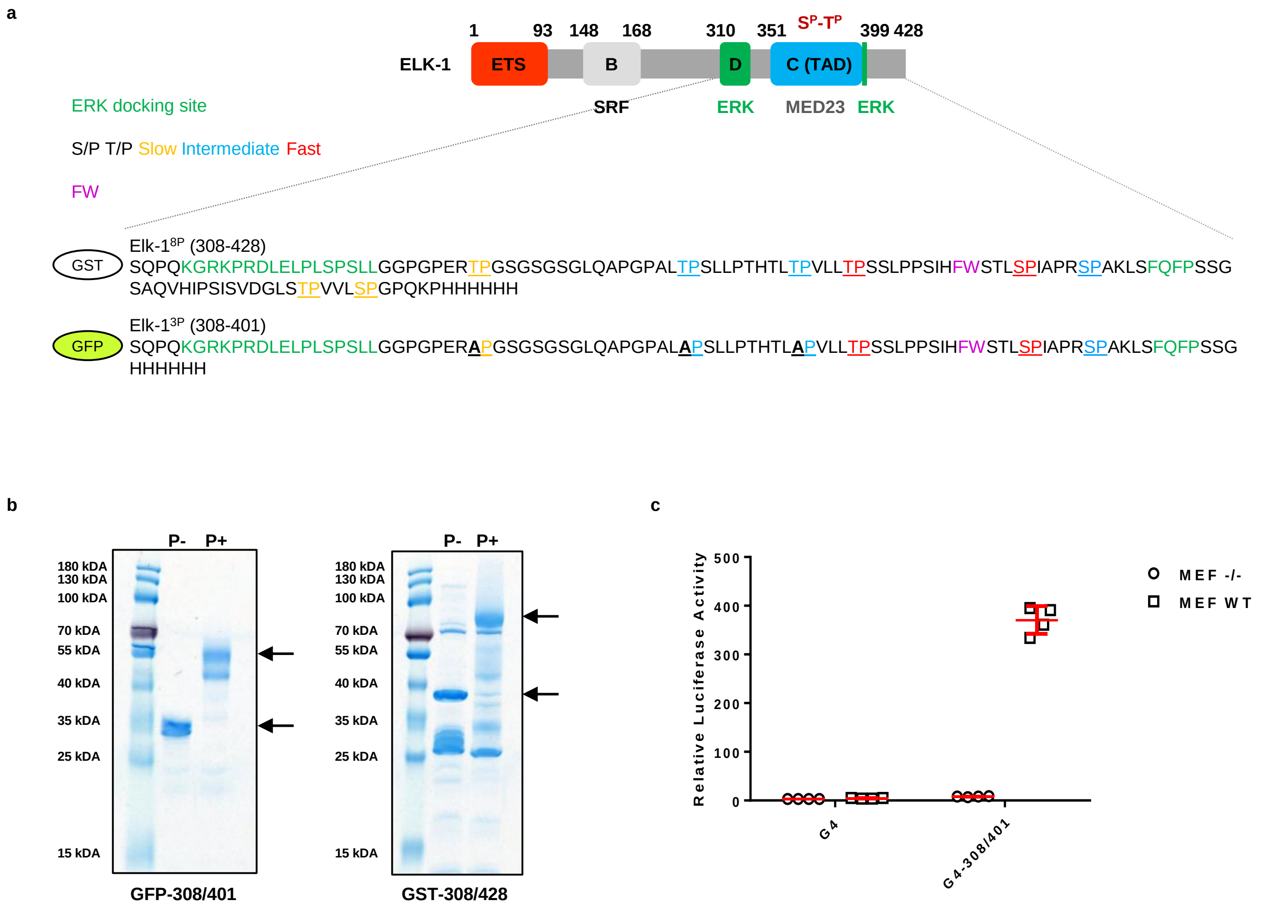

Supplementary Fig. 5: **Phosphorylation of Elk-1. a,** Sequence of human Elk-1 TAD. S/T-P phosphorylation sites are highlighted according to ERK modification rates (fast: red; intermediate: cyan and slow: orange). The D-box and FQFP ERK docking sites are shown in green and the central FW-MED23 binding motif in purple. The two constructs used in this study are indicated (GST-Elk-1^{8P} (308-428) and GFP-Elk-1^{3P} (308-401)). The GFP-Elk-1^{3P} (308-401) construct used for Cryo-EM is phosphorylated at three positions (T368, S383 and S389). For other phosphorylation sites, the serine and threonine residues were either replaced by alanine residues (T336, T353 and T363) or not included (T417 and S422). The equivalent construct was also fused to Gal4 DNA binding domain for transcriptional activity. **b,** Phosphorylation profiles of GFP-Elk-1^{3P} (308-401) and GST-Elk-1^{8P} (308-428). Recombinant purified proteins were separated by Phos-tag SDS-PAGE and visualized by Coomassie Blue staining. Molecular weight marker (in kDa) is indicated on the left. P-: non-phosphorylated; P+: phosphorylated. **c,** Transcriptional activity of Elk-1^{3P} (308-401). MED23$^{-/-}$ MEFs and MEFs$^{+/+}$ were transfected with a 5 × Gal4-E1B-TATA-luciferase reporter construct and a plasmid encoding either Gal4 alone (G4) or Gal4-Elk-1^{3P} (308-401) as indicated. Firefly luciferase activity was normalized to Renilla luciferase activity. Data are representative of three independent experiments and are presented as mean values ±SD

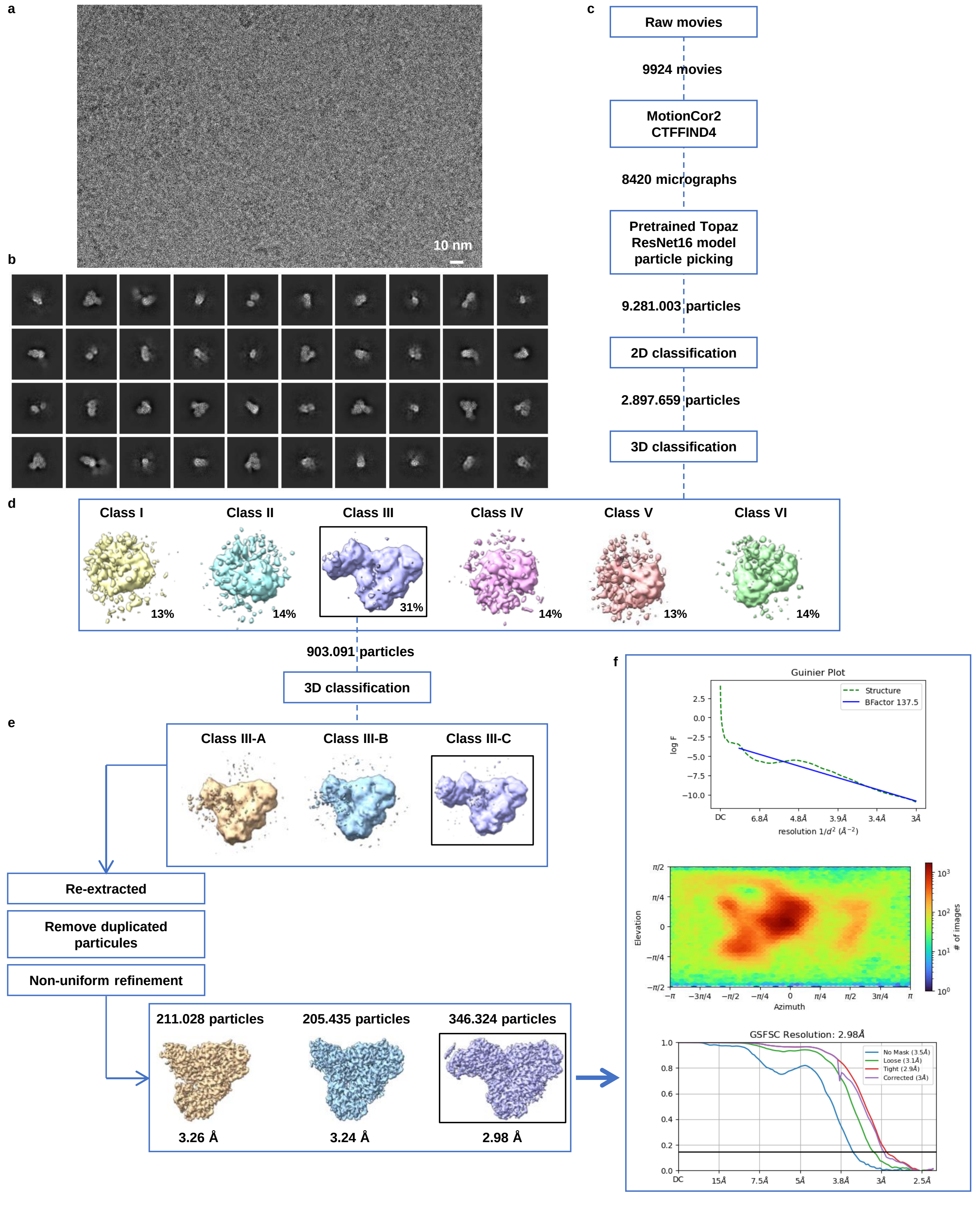

<u>Supplementary Fig. 6:</u> MED23^{Elk-1} cryo-EM data collection and processing. a, Representative cryo-electron micrograph of MED23^{Elk-1} sample. **b,** class averages after reference-free 2D classification generated with cryoSparc. Box size corresponds to 300 Å. **c,** Processing tree. The number of selected particles for each step is indicated. **d,** 903.091 particle images were assigned to a 3D-class (class III) showing density for MED23 entity and **e,** further subjected to focused classification (class III-A, III-B and III-C). 3D-classes are color-coded. A final reconstruction at 3.0 Å resolution was obtained by non-uniform refinement. The two classes (III-A and III-B) that showed disordered or degraded HR1 were also submitted to non-uniform refinements with a global resolution of ~3.3 Å. **f**, Guinier plot used for local B-factor estimation, angular distribution plot after reconstruction with cryoSparc and Fourier shell correlation plot for the focused refined map.

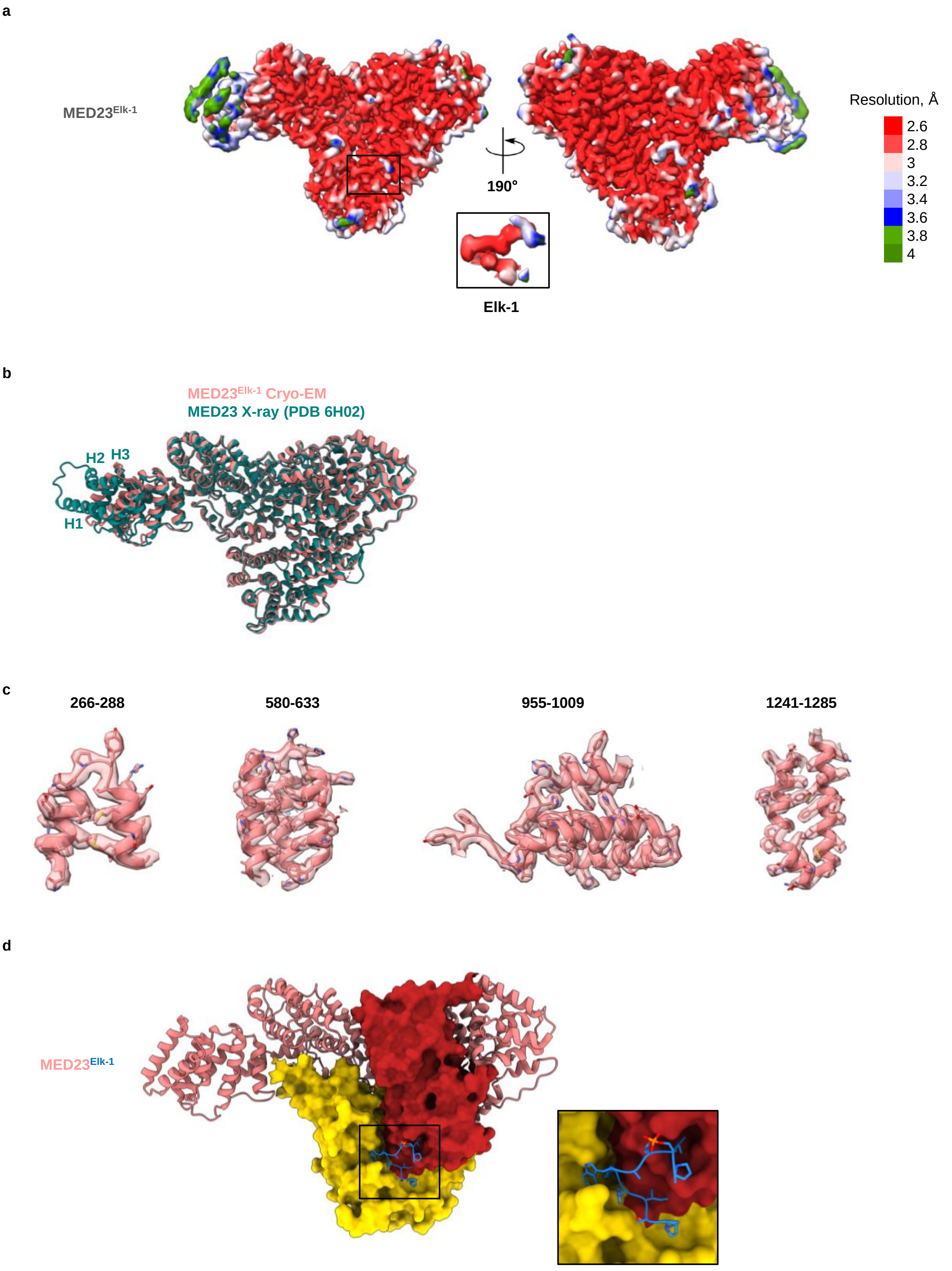

<u>Supplementary Fig. 7:</u> 3 Å resolution Cryo-EM map of MED23^{Elk-1}. a, Local resolution of MED23^{Elk-1} with a 0.5 threshold for local FSC resolution estimation calculated in cryoSparc. The cryo-EM density surface is colored according to the resolution estimates, ranging from 2.6 Å (red) to 4 Å (green) depicted from the concave view and rotated by 190° (convex view). MED23 is well resolved below 3 Å, except for small region in loops and HR1 region. Local resolution of Elk-1 (374-384) is also shown in the expanded view indicated by a black box. **b,** Superimposition of MED23^{Elk-1} (in light coral) with MED23 crystal structure (PDB 6H02 in teal). Residues 1-50 that contains the three N-terminal helices (H1 to H3) only showed blobs of density and thus have not been included in the model. **c,** Map-model fit of selected regions in surface representation for MED23^{Elk-1}. Side chains are represented in stick. **d**, MED23-Elk-1 interface. Elk-1 binding site on MED23 is observed at the interface between HR2 and HR3 regions. MED23 colored in light coral is represented in ribbons, HR2 and HR3 in surface and color-coded as in Supplementary Fig. 4. Close-up view of the interface is also shown and indicated by a black box with Elk-1 (374-384) in stick representation and colored in blue.

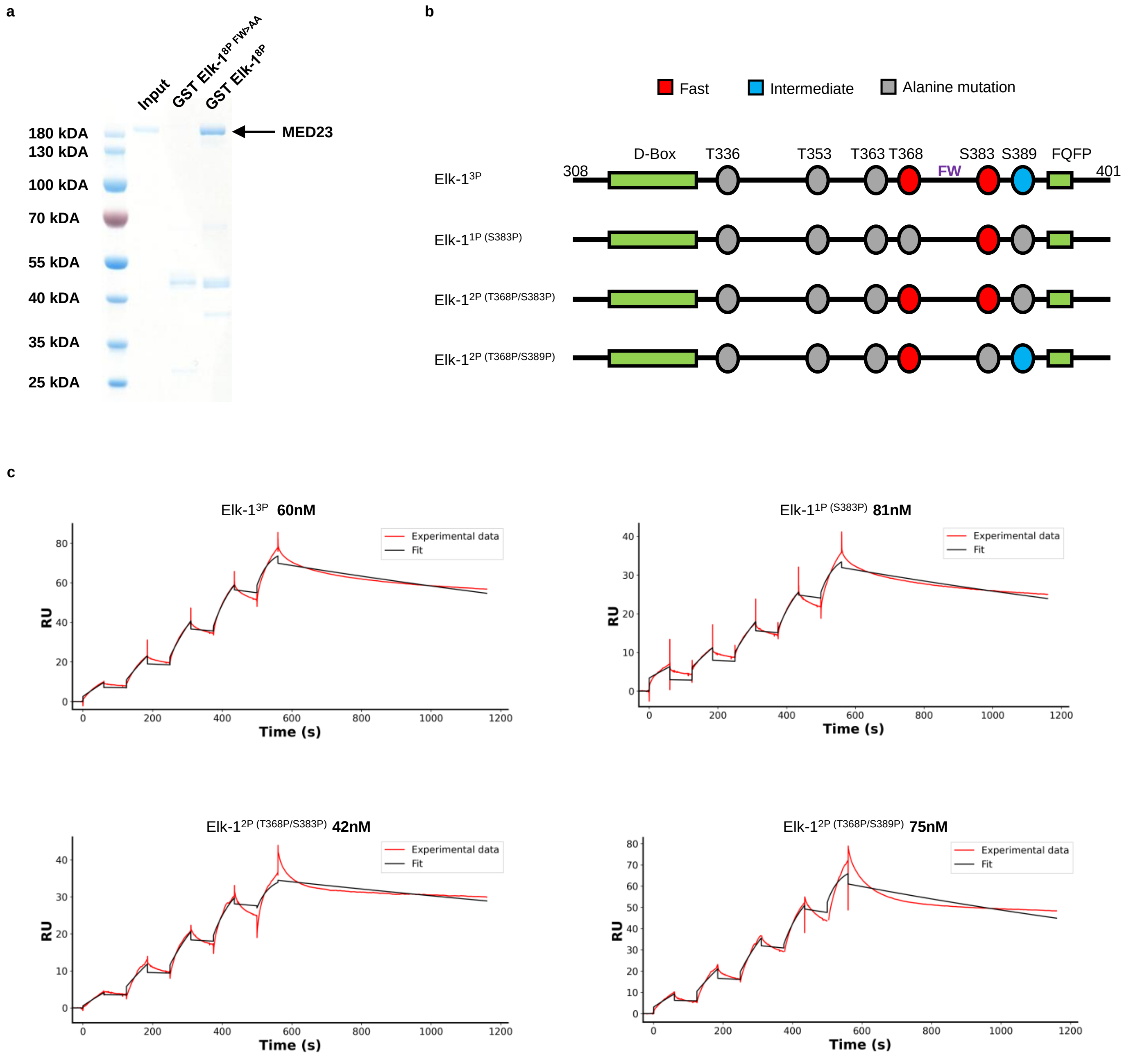

Supplementary Fig. 8: Contribution of Elk-1 phosphorylation to MED23 binding. a, MED23 binding is disrupted by Elk-1 FW mutation. GST Elk-1^{8P} (308-428) and GST Elk-$1^{8P\ FW>AA}$ (308-428) were immobilized on GST-Trap agarose and incubated with recombinant MED23. Bound proteins were eluted with Laemmli sample buffer, resolved by SDS-PAGE, and visualized by Coomassie Blue staining. Molecular weight marker (in kDa) is indicated on the left. Input 1/40. **b,** Outline of analyzed Elk-1^{3P} mutants. Fast (red) and intermediate (blue) S/T-P phosphorylation sites, kinase docking motifs (green) and the FW residues (purple) are shown. **c,** Single-cycle kinetic analysis determined by surface plasmon resonance of the interaction of Elk-1^{3P} derivatives with immobilized MED23. Binding curves were fitted using Langmuir (1:1 binding) model.

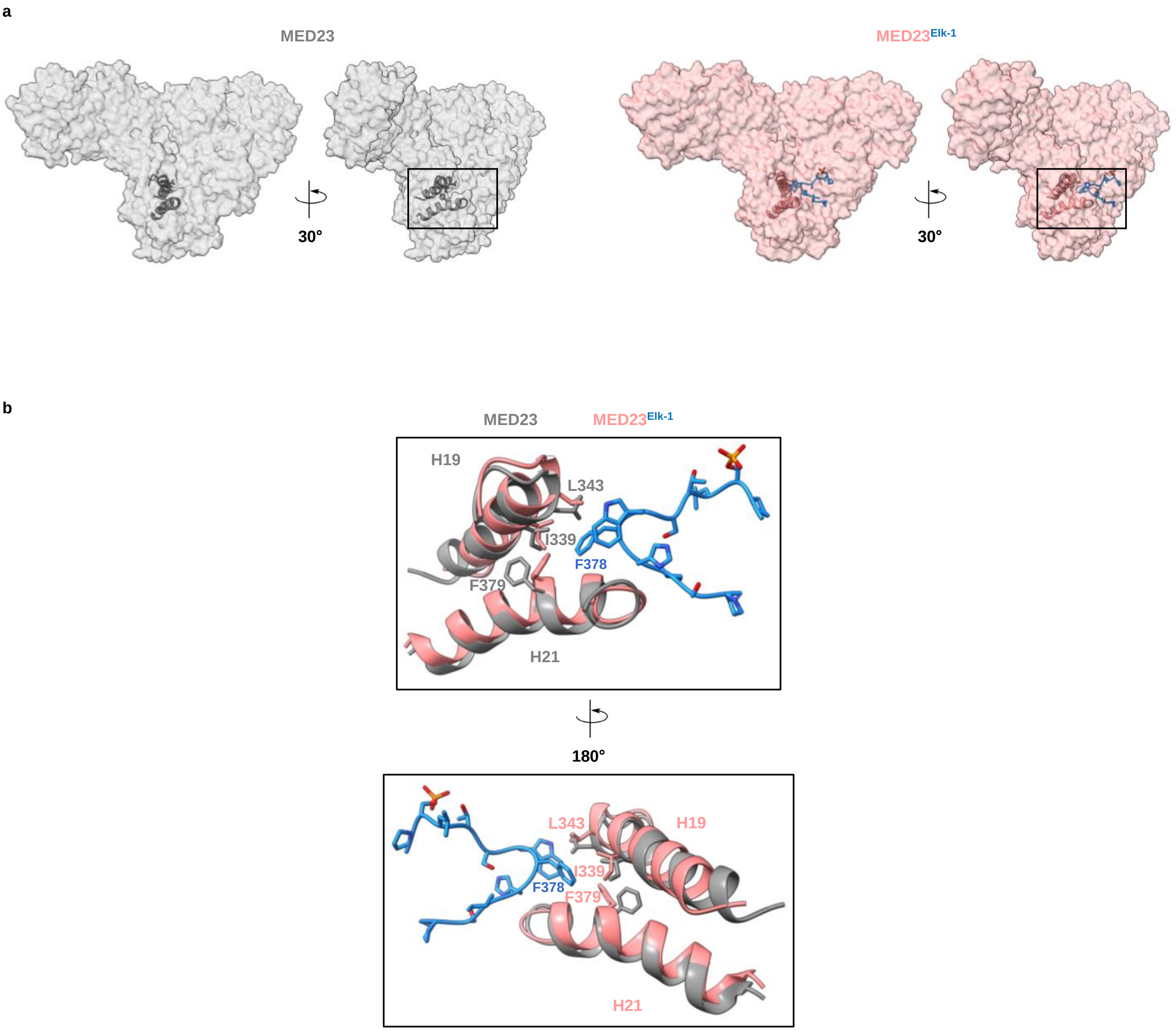

<u>Supplementary Fig. 9:</u> Global movement of helix H19 in HR2 necessary for MED23 to accommodate F378-Elk-1. a, two concave views rotated by 30° of MED23 (in gray) and MED23$^{Elk\text{-}1}$ (in light coral) structures in transparent surface representation highlighting H19 and H21 helices in cartoon representation, related to Figs. 1, 2, 3 and 4. **b,** Close-up view of H19 and H21. MED23 side chains discussed throughout the text are represented in stick and color-coded to label amino acid numbers. MED23 helices are shown as ribbons. Two views are shown rotated by 180°. Elk-1 (374-384) in stick representation colored in blue with F378 indicated.

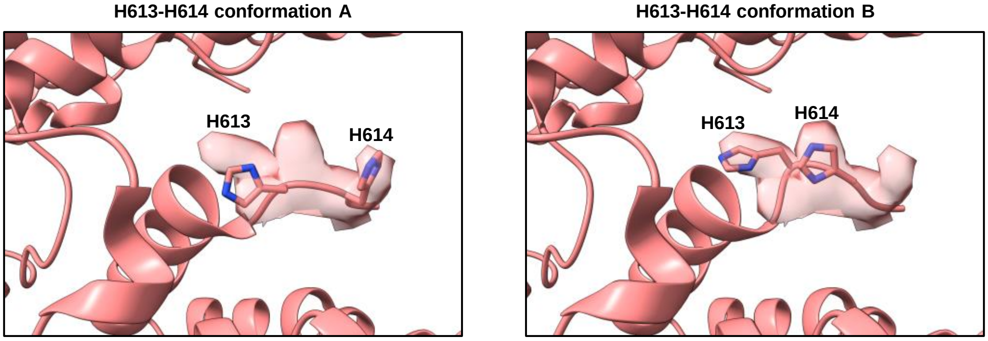

<u>Supplementary Fig. 10:</u> H613 and H614 double conformations. Related to Fig. 5. Map-model fit of H613 and H614 from MED23$^{Elk\text{-}1}$ cryo-EM map (in light coral) in surface representation. The two side chains conformations are indicated and represented in stick. Region 666-677 of MED23 was omitted for clarity.

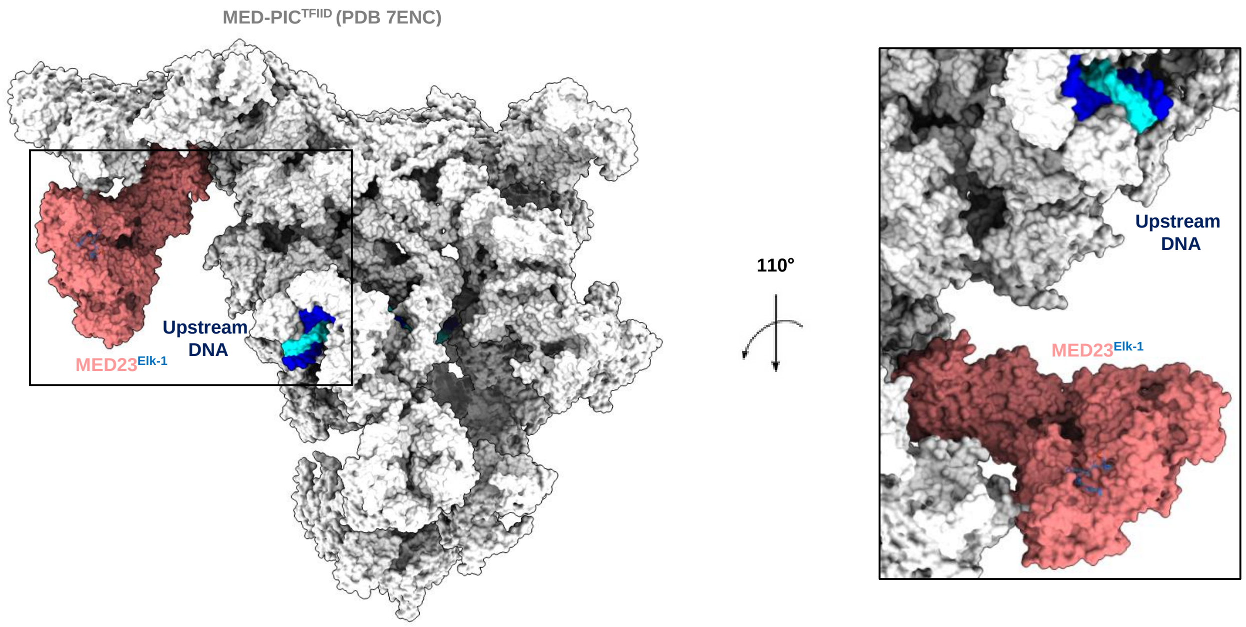

<u>Supplementary Fig. 11:</u> Structure of Mediator-bound preinitiation complex. Superimposition of MED-PIC^{TFIID} (PDB 7ENC) cryo-EM structure colored in light gray with MED23^{Elk-1} cryo-EM structure (this study) colored in light coral and surface representation. Upstream DNA template and non-template strands are in dark and light blue. Expanded view is indicated by a black box and rotated by 110°. Elk-1 is orientated towards the upstream DNA, ideally suited to interact with upstream regulatory sequences.

**Antibody**	**Host**	**Compagny (Reference)**	**Use**
MED23	Mouse	BD Pharmigen # 550429	WB 1:1000
Egr1	Rabbit	Cell Signaling # 15F7	WB 1:1000
Beta-Actin	Mouse	Invitrogen# BA3R-HRP	WB 1:4000

**Plasmid**	**Origin**
pFastBac	Thermo
pFastBac Dual	Thermo
FC-550A-1	SBI
PIN510A-1	SBI
FC200PA-1	SBI
pGEX6P1	Novagen
pGEX4T1	Novagen
pColaDUET	Novagen
pET-GFPm	Genscript-This study
pcDNA3-Gal4	[58]
pFastBac MED23	[40]
pFastBac MED23 G382F	This study
pFastBac Dual BAP MED23-BIRA	This study
FC550A-1-MED23	This study
FC550A-1-MED23-G382F	This study
PIN510A-1 MED23	This study
PIN510A-1 MED23-G382F	This study
pGEX6P1-Elk1^{8P} (308-428)-6HIS	This study
pGEX6P1-Elk1$^{8P\ FW>AA}$ (308-428)-6HIS	This study
pGEX4T1-Elk1^{3P} (308-401)-6HIS	This study
pGEX4T1-Elk1^{S383P} (308-401)-6HIS	This study
pGEX4T1-Elk1$^{T368P/S383P}$ (308-401)-6HIS	This study
pGEX4T1-Elk1$^{T368P/S389P}$ (308-401)-6HIS	This study
pcDNA3-Gal4 Elk1^{3P} (308-401)	This study
pcDNA3-Gal4 Elk1^{8P} (308-428)	This study
pcDNA3-Gal4-13S (121-223)	[58]
pcDNA3-Gal4	[58]
pET-GFPm-Elk1^{3P} (308-401)-6HIS	Genscript-This study
pGEX4T3-HA-ERK2-GOF	Addgene#53200
pGEX4T1-3XHA-MEK1DD	Addgene#47576
pColaDUETMBP-ERK2-GOF/MEK1DD	This study
pCMV Elk-1	[33]
pG5E1B-LUC	Promega
pCMV Renilla	Promega

**Miscellaneous**	**Origin**
GST-Trap	Chromotek # sta-20
mPAGE 4-20% Bis-Tris precast gel	Genscript # MP42G15

Supplementary Table 1. Plasmids, antibodies and reagents.
