# Supplementary material for: Structural basis of human Mediator recruitment by the phosphorylated transcription factor Elk-1
Source: Nat Commun. 2025 Apr 22;16:3772. doi: 10.1038/s41467-025-59014-8 (PMC12015215; doi:10.1038/s41467-025-59014-8)
Supplement: Supplementary file 2 — Reporting Summary [file 41467_2025_59014_MOESM2_ESM.pdf]

nature portfolio

Corresponding author(s): Vincent Villeret

Last updated by author(s): 2025/03/20

Reporting Summary

Nature Portfolio wishes to improve the reproducibility of the work that we publish. This form provides structure for consistency and transparency in reporting. For further information on Nature Portfolio policies, see our Editorial Policies and the Editorial Policy Checklist.

Statistics

For all statistical analyses, confirm that the following items are present in the figure legend, table legend, main text, or Methods section.

n/a	Confirmed	
[ ]	[x]	The exact sample size (*n*) for each experimental group/condition, given as a discrete number and unit of measurement
[ ]	[x]	A statement on whether measurements were taken from distinct samples or whether the same sample was measured repeatedly
[x]	[ ]	The statistical test(s) used AND whether they are one- or two-sided<br>*Only common tests should be described solely by name; describe more complex techniques in the Methods section.*
[x]	[ ]	A description of all covariates tested
[x]	[ ]	A description of any assumptions or corrections, such as tests of normality and adjustment for multiple comparisons
[ ]	[x]	A full description of the statistical parameters including central tendency (e.g. means) or other basic estimates (e.g. regression coefficient) AND variation (e.g. standard deviation) or associated estimates of uncertainty (e.g. confidence intervals)
[x]	[ ]	For null hypothesis testing, the test statistic (e.g. *F*, *t*, *r*) with confidence intervals, effect sizes, degrees of freedom and *P* value noted<br>*Give P values as exact values whenever suitable.*
[x]	[ ]	For Bayesian analysis, information on the choice of priors and Markov chain Monte Carlo settings
[x]	[ ]	For hierarchical and complex designs, identification of the appropriate level for tests and full reporting of outcomes
[x]	[ ]	Estimates of effect sizes (e.g. Cohen's *d*, Pearson's *r*), indicating how they were calculated

Our web collection on statistics for biologists contains articles on many of the points above.

Software and code

Policy information about availability of computer code

Data collection	Western blot images were acquired using Amersham ImageQuant 800 imaging system.
Data analysis	UCSF Chimera X 1.7.1, Coot 0.9, PHENIX 1.18, cryoSPARC 2.14.2, Molprobity 4.5.1. Graphs were plotted and analyzed using Graphpad Prism 8, SPR data were analyzed using Biacore T200 Evaluation Software 3.0

For manuscripts utilizing custom algorithms or software that are central to the research but not yet described in published literature, software must be made available to editors and reviewers. We strongly encourage code deposition in a community repository (e.g. GitHub). See the Nature Portfolio guidelines for submitting code & software for further information.

Data

Policy information about availability of data

All manuscripts must include a data availability statement. This statement should provide the following information, where applicable:

- Accession codes, unique identifiers, or web links for publicly available datasets
- A description of any restrictions on data availability
- For clinical datasets or third party data, please ensure that the statement adheres to our policy

Cryo-EM maps and atomic coordinates have been deposited with the Electron Microscopy Data Bank and Protein Data Bank. MED23 (PDB 9F76, EMD-50247) and MED23-Elk-1 complex (PDB 9F6Y, EMD-50242)

Research involving human participants, their data, or biological material

Policy information about studies with human participants or human data. See also policy information about sex, gender (identity/presentation), and sexual orientation and race, ethnicity and racism.

Reporting on sex and gender	n/a
Reporting on race, ethnicity, or other socially relevant groupings	n/a
Population characteristics	n/a
Recruitment	n/a
Ethics oversight	n/a

Note that full information on the approval of the study protocol must also be provided in the manuscript.

Field-specific reporting

Please select the one below that is the best fit for your research. If you are not sure, read the appropriate sections before making your selection.

☒ Life sciences ☐ Behavioural & social sciences ☐ Ecological, evolutionary & environmental sciences

For a reference copy of the document with all sections, see nature.com/documents/nr-reporting-summary-flat.pdf

Life sciences study design

All studies must disclose on these points even when the disclosure is negative.

Sample size	No statistical methods were used to predetermine sample size. Sample sizes were chosen for the different experimental approaches based on the technical difficulty and throughput of the individual assays, the chosen sample sizes are consistent with previous publications. All biochemical and cell culture experiments were replicated three or more times. Structural data was collected on independently prepared samples.<br>For CryoEM image data, the number of images included in the analysis was selected to maximize resolution under the chosen experimental conditions.
Data exclusions	No data were excluded from the analyses except for application of standard CryoEM protocols (2D image clustering) for elimination of non-particle images included in the initial set of images obtained by automated particle selection.
Replication	All attempts at replication were successful, at least three repetitions for biochemical assays were performed. Cryo-EM single particle analysis inherently relies on averaging over a large number of independent observations. For cell culture, representative figure of at least 3 independent experiments.
Randomization	There was no allocation of test subjects for any experiments, thus randomization was not applicable to our study.
Blinding	Data analyses were performed by unbiased software programs/algorithms. Blinding was therefore not applicable to our study.

Reporting for specific materials, systems and methods

We require information from authors about some types of materials, experimental systems and methods used in many studies. Here, indicate whether each material, system or method listed is relevant to your study. If you are not sure if a list item applies to your research, read the appropriate section before selecting a response.

Materials & experimental systems

n/a	Involved in the study	
☐	☒	Antibodies
☐	☒	Eukaryotic cell lines
☒	☐	Palaeontology and archaeology
☒	☐	Animals and other organisms
☒	☐	Clinical data
☒	☐	Dual use research of concern
☒	☐	Plants

Methods

n/a	Involved in the study	
☒	☐	ChIP-seq
☒	☐	Flow cytometry
☒	☐	MRI-based neuroimaging

Antibodies

Antibodies used	MED23 Mouse BD Pharmigen # 550429 WB 1:1000<br>Egr1 Rabbit Cell Signaling # 15F7 WB 1:1000<br>Beta-Actin Mouse Invitrogen# BA3R-HRP WB 1:4000
Validation	We used each antibody following to the application stated in the manufacturer's websites.

Eukaryotic cell lines

Policy information about cell lines and Sex and Gender in Research

Cell line source(s)	MED23 -/- and MED23 +/+ MEFs (Stevens et al. 2002 PMID: 11934987) were cultured in DMEM containing 10% FBS.<br><br>ExpiSF9 cells (Thermo A35243) were cultured according to manufacturer’s instructions.
Authentication	Authentication of the MEFs KO cell line was performed by Wester-blot. The insect cells were authenticated by the manufacturer.
Mycoplasma contamination	ExpiSF9 cell line was not tested for mycoplasma contamination. MEFs cell lines were regularly tested for mycoplasma contamination and were negative.
Commonly misidentified lines<br>(See ICLAC register)	No commonly misidentified cell lines were used.

Plants

Seed stocks	n/a
Novel plant genotypes	n/a
Authentication	n/a
