# Supplementary figures and images for: Structural basis of human Mediator recruitment by the phosphorylated transcription factor Elk-1
Source: Nat Commun. 2025 Apr 22;16:3772. doi: 10.1038/s41467-025-59014-8 (PMC12015215; doi:10.1038/s41467-025-59014-8)

## Slide 1
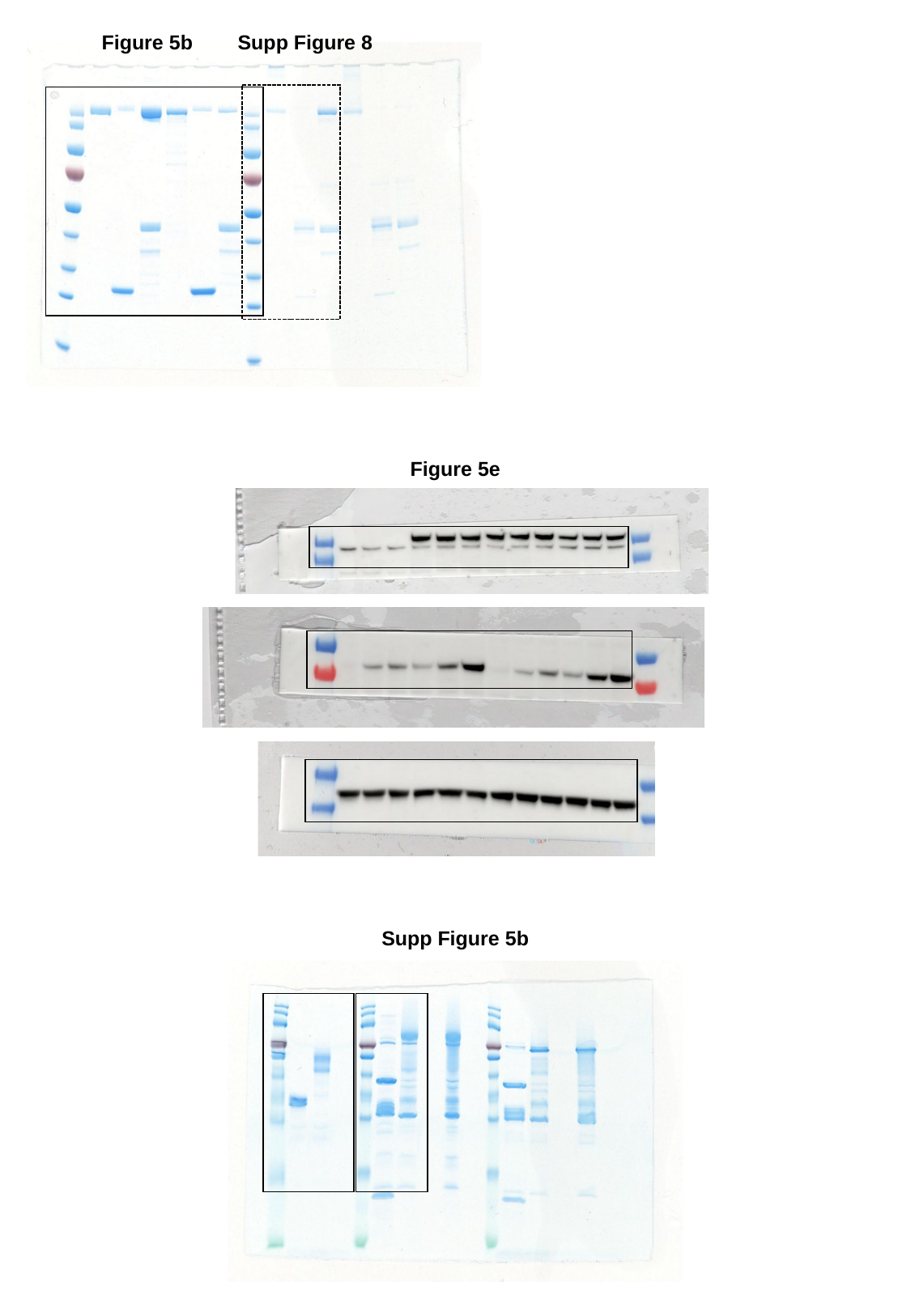

Figure 5b
Supp Figure 8
Figure 5e
Supp Figure 5b

Supplement: Supplementary file 4 — Source data [file 41467_2025_59014_MOESM4_ESM.zip › 503715_2_related_ms_10529099_stmfh9.pptx]
